# Medium-Chain Triglyceride Oil and Dietary Intervention Improved Body Composition and Metabolic Parameters in Children with Glycogen Storage Disease Type 1 in Jordan: A Clinical Trial

**DOI:** 10.3390/foods13071091

**Published:** 2024-04-02

**Authors:** Hadil S. Subih, Reem A. Qudah, Sana Janakat, Hanadi Rimawi, Nour Amin Elsahoryi, Linda Alyahya

**Affiliations:** 1 Department of Nutrition and Food Technology, Faculty of Agriculture, Jordan University of Science and Technology, P.O. Box 3030, Irbid 22110, Jordan; reem_qudah9570@hotmail.com (R.A.Q.); jana@just.edu.jo (S.J.); 2 Royal Medical Services, P.O. Box 712996, Amman 11171, Jordan; hanadi_rimawi@yahoo.com; 3 Department of Nutrition, Faculty of Pharmacy and Medical Sciences, University of Petra, Amman 11196, Jordan; nour.elsahoryi@uop.edu.jo; 4 School of Pharmaceutical Sciences, Universiti Sains Malaysia, Gelugor 11800, Malaysia; lalyahya86@student.usm.my

**Keywords:** glycogen storage disease type 1, MCT oil, hypoglycaemia, growth parameters, uncooked corn starch, diet

## Abstract

Glycogen storage diseases (GSDs) are a group of carbohydrate metabolism disorders, most of which are inherited in autosomal recessive patterns. GSDs are of two types: those that have to do with liver and hypoglycaemia (hepatic GSDs) and those that are linked to neuromuscular presentation. This study aims to assess the impact of dietary intervention, including medium-chain triglyceride (MCT) oil, on anthropometric measurements, body composition analysis and metabolic parameters among Jordanian children and is expected to be the first in the country. A sample of 38 children with glycogen storage disease type 1 (GSD-1) (median age = 6.4 years) were on a diet that included uncooked cornstarch therapy and a fructose-, sucrose- and lactose-restricted diet. Patients started to take MCT oil along with the prescribed diet after the first body composition test. Patients’ nutritional status was re-evaluated three months later. The study results show that the percentage of patients who suffered from hypoglycaemia at the beginning of the study decreased significantly from 94.7% to 7.9% (*p* < 0.0001). The serum levels of triglycerides, cholesterol, uric acid and lactate decreased significantly after three months of intervention (100–71.1%, 73.7–21.1%, 97.4–52.6% and 94.7–18.4%, respectively). In contrast, there was no statistical difference in neutrophil count. Regarding clinical parameters, liver span was significantly reduced from (16.01 ± 2.65 cm) to (14.85 ± 2.26 cm) (*p* < 0.0001). There were significant improvements in growth parameters, including height-for-age and BMI-for-age for children aged ≥2 years (*p* = 0.034 and *p* = 0.074, respectively). Significant improvements in skeletal muscle mass and bone mineral content were also noticed at the end of the trial (*p* ≤ 0.05). In conclusion, medium-chain triglyceride therapy is found to improve biochemical and growth parameters in children with GSD-1 in Jordan.

## 1. Introduction

Glycogen storage diseases (GSDs) are a group of carbohydrate metabolism disorders, most of which are inherited in autosomal recessive patterns [1]. There are two sorts of GSDs: those that have to do with liver and hypoglycaemia (hepatic GSDs) and those that are linked to neuromuscular presentation [2,3]. Based on the type of enzyme defect, about 16 types have been discovered and numbered from 0 to 15 [1,4]. Subsequently, the protocols of treatment and management are different, but nutrition intervention is still the cornerstone of management for all types of GSDs [5]. Basically, GSD-1, or von Gierke’s disease, is one of the most common types of glycogen storage diseases. It is an autosomal recessive inherited disorder of carbohydrate metabolism in which glycogen cannot be metabolised and converted back into glucose, which results in the buildup of glycogen and fat in the liver and kidneys, which, in turn, causes enlargement of both organs, i.e., hepatomegaly and renomegaly, respectively, and developmental delay [6,7,8,9,10]. GSD-1 is caused by a deficiency of one of two enzymes: either glucose-6-phosphatase (GSD-1a), which is expressed in the liver, kidney and intestine [11] or glucose-6-phosphate translocase (G6PT) (GSD-1b), which is expressed in leukocytes in addition to the previous sites [7,12]. Among these two major subtypes of glycogen storage disease type 1, GSD-1a is the more common, with 80% of patients with GSD-1 being classified as type 1a [1,4,13,14,15]. The overall incidence is approximately 1 in 100,000 live births [9,16,17,18,19] in Caucasians, Hispanics and Asians. It is more common in Ashkenazi Jews, with an incidence of 1 in 20,000 [8].

In Jordan, the total number of patients diagnosed with type 1 is around 40 patients, according to Dr Mu’men Aqeel at the metabolic clinic at Queen Rania Al Abdullah Hospital for Children. GSD-1 can be diagnosed clinically from biochemical abnormalities shown in blood tests and the familial history of the disease, or it can be diagnosed based on a liver biopsy, which reveals the accumulated amounts of glycogen and fat within the hepatocytes [8]. According to certain enzyme deficiencies, the process of glycogenolysis and gluconeogenesis is compromised [12,20], which causes fasting hypoglycaemia and usually appears between the ages of three and four months of life or immediately after birth [6,9], especially during long periods of sleep or interrupted feeding patterns due to illness [1,21,22]. In addition, other metabolic imbalances can appear, such as hyperlipidaemia, lactic acidosis, hyperuricemia, hepatomegaly and renomegaly [22,23]. Patients with GSD-1 have doll-like faces with fatty cheeks, relatively thin extremities, distended abdomens, short stature and developmental delay [1] and, in GSD-1b only, neutrophil impairment with recurrent infections that may progress to inflammatory bowel disease (IBD) [1,11,24].

The other method of choice for diagnosis is genetic testing, which additionally confirms the subtypes of GSD-1, type 1a (*G6PC* gene) and type 1b (*SLC37A4* gene) [8]. It is essential to note that perfect and early diagnosis, in addition to strict adherence to dietary guidelines, can decrease the severity of disease complications. Hence, a relatively normal development pattern and good quality of life can be obtained [21,25,26]. Patients who do not receive treatment may suffer from long-term complications, such as hepatic adenomas, renal disease, pancreatitis and changes in brain function [19,27]. Some may have anaemia and rickets [6,13,28,29], while others may develop systemic hypertension and carcinoma prognosis of the hepatic adenoma and gout due to high levels of uric acid [16,30,31] and bleeding diathesis due to platelet dysfunction [8,29,30]. However, these complications can be minimised by adequate management and controlled metabolic profiles [2,15].

Studies conducted previously focused mainly on strategies for preventing life-threatening hypoglycaemia, thus neglecting the short- and long-term metabolic complications of the disease [18,32,33,34,35,36], which affect the quality of life and survival rates among patients with GSD [1,34,37,38]. The efficacy of the dietary treatment can be assessed in several ways.

1. Biochemical blood tests, including blood glucose levels, triglycerides, cholesterol, lactic acid and uric acid, are carried out to determine to what extent a patient sticks to the prescribed diet.
2. Abdominal ultrasound to measure the volume of the liver and kidneys to assess whether or not there is an enlargement.
3. Dual-energy X-ray absorptiometry (DEXA) to assess bone mineral content and vitamin D status.
4. Growth parameters, including body weight and height.
5. Assessment of adherence to diet schedule [36,38] and check-up on proper consumption of low glycaemic carbohydrates to avoid obesity [39].

Usually, the protocol of follow-up and monitoring is best repeated every three to four months (except for DEXA scans, once a year), in particular when patients are in their stable state. However, patients who are decompensated need more repeated observation and checking [40]. It is essential to monitor blood glucose levels at home using glucometers. Parents should be instructed on how and when to check their children’s glucose levels so that they can avoid hypoglycaemic episodes [32]. In this study, our hypothesis indicated that dietary and MCT oil intervention will improve the metabolic profile and growth parameters of paediatric patients with GSD type 1.

## 2. Materials and Methods

### 2.1. Study Design

This is a clinical trial study that was conducted at Queen Rania Al Abdullah Hospital for Children (QRAH) in King Hussein Medical City, Amman, Jordan, from April to November 2019. The study was conducted according to the guidelines laid down in the Declaration of Helsinki, and all procedures involving human subjects/patients were approved by the Institutional Research Board (IRB) of Jordan University of Science and Technology (JUST), which was obtained in session number 28/126/2019. Written informed consent was obtained from all patients’ parents. Patients were met at the metabolic clinic at QRAH, as it is considered the major centre for rare inherited metabolic disorders, including GSD-1.

### 2.2. Patient Recruitment

A sample of 38 children diagnosed with GSD-1 (1a: *n* = 11, 1b: *n* = 27) and with a median age of 6.4 years (range: 1.5–15.5 years) were recruited from the metabolic clinic during routine follow-up visits. The median age at diagnosis was five months. Patients’ data, including brief disease histories, biochemical blood results, abdominal ultrasound reports and prescribed medications, were collected from the electronic records system used by the hospital. Diagnosis of the disease was confirmed by several methods mentioned in the following section headed ‘inclusion and exclusion criteria’. All patients were already on uncooked cornstarch (UCCS) therapy and a fructose-, sucrose- and lactose-restricted diet. The children were recommended to take the GSD diet at diagnosis, while they were asked to take the MCT at the first follow up session after accepting to participate in the study. So, the period the children were on the GSD diet was different depending on the date of diagnosis. MCT oil was added to the GSD traditional diet after the first body composition (InBody) test. At baseline, before the MCT oil intervention, the laboratory results, anthropometric measurements, and body composition analyses were collected and subsequently re-evaluated three months later following the MCT oil intervention. Consent forms were obtained from the parents of the patients at the beginning of the study.

### 2.3. Inclusion and Exclusion Criteria

The inclusion criteria were based on the method of the disease diagnosis. All patients who were diagnosed based on the following criteria were included in the study.

1. Positive family history of the condition as long as GSD has an autosomal recessive inheritance pattern.
2. Biochemical findings, including hypoglycaemia, hyperlipidaemia, lactic acidosis, hyperuricemia and neutropenia, which are consistent with 1b only can be used as a tool to distinguish between the two subtypes, 1a and 1b.
3. Physical considerations such as a rounded doll face, distended abdomen and short stature.
4. Liver biopsy findings that show huge amounts of glycogen within the hepatocytes.
5. Genetic testing that rules out the glycogen storage disease panel so that either mutated gene can be obtained: *G6PC* gene for 1a or *SLC37A4* gene for 1b.

Patients with infectious or malignant causes of hepatomegaly or other storage disorders were excluded from the study.

### 2.4. Questionnaire

The questionnaire was validated by circulating it to three faculty members in different institutions, and all modifications were made. It is composed of four major parts: socio-demographic information, health-related information, including the child’s height, age at diagnosis and vitamin deficiencies, dietary intake assessment and lifestyle behaviours, including sleeping hours per day and whether parents were smokers and how frequently.

### 2.5. Anthropometry and Body Composition Analysis

Parents were instructed to dress their children in light clothing and to try to get their children to empty their bladders before every visit. Height was measured barefoot using the portable DETECTO^®^ stadiometer (Webb City, MO, USA). Body weight was measured using the bioelectrical impedance analysis (InBody^®^ 570, Cerritos, CA, USA) body composition analyser. The InBody^®^ device is connected to a printer that prints out a result sheet specific to each child. The researcher then discussed the result sheet with the child and parents and focused on anthropometric measurements, including height, weight and body mass index (BMI), which was calculated based on the BMI Percentile Calculator for Children and Teenagers aged 2–19 years, and body composition analysis, including skeletal muscle mass (SMM), body fat mass (BFM), percentage body fat (PBF) and bone mineral content (BMC) was carried out. These data were obtained at baseline (before MCT oil intervention) and three months later during follow-up visits. Additionally, the researcher reviewed the diet schedule, which is kept by parents as a hard copy, and explained the importance of adherence to the nutritional guidelines in keeping the body composition and anthropometry relatively within normal ranges. The World Health Organization (WHO) growth charts were used for patients aged <2 years, whereas the Centers for Disease Control and Prevention (CDC) growth charts were used for patients aged ≥2 years in order to assess their growth and development [41]. The researcher encouraged the children with a 450 mL mug with a coloured lid and a straw (Table 1).

### 2.6. Dietary Intervention

Patients with GSD-1 were counselled, and recommendations were made based on van Calcar (38), who postulated the proper dietary guidelines as follows.

- First, energy needs should be distributed throughout the day according to height, body weight and physical activity (complex carbohydrates 60–70%, proteins 15–20% and fats less than 30%).
- Second, milk formulas used must not contain sucrose, lactose or fructose.
- Third, infants who are breastfed are allowed to continue unless they are metabolically uncontrolled. Their feeding intervals must be every two to three hours, day and night, to keep blood glucose levels stable.
- Fourth, the diet should be lactose-, fructose- and sucrose-free or intake is to be severely restricted.
- Fifth, measured amounts of UCCS, according to the patient’s age and body weight. Between 1.75 and 2.5 g/kg of body weight every 4–6 h for children over two years and 1.6 g/kg of body weight every 3–4 h for young children from six months up to two years old, to be administered with water or non-acidic fluids. Acidity will break down the glycosidic bonds by which starch loses its performance at a ratio of 1:2, which gives a slow release of glucose to maintain euglycemia (≥70 mg/dL or 4 mmol/L).
- Sixth, MCT oil (0.16–0.44 g/kg and day for 32–40 months) is prescribed starting from the day of diagnosis based on the physician’s prescription.
- Finally, sucrose and lactose-free vitamin and mineral supplementations are prescribed by the metabolic physician to ensure optimal nutrition intake. (This diet is not nutritionally well-balanced regarding the amounts of certain vitamins and minerals. Therefore, patients are recommended to consume multivitamins and minerals, especially calcium and vitamin D_2_.).

### 2.7. Clinical Assessment Sheet 

Parents and their children were asked for additional personal information essential for both the study and the physician’s progress note, such as the child’s date of birth, age at diagnosis and degree of consanguinity, as well as diagnosis modality (clinical examination, family history, liver biopsy, enzyme assay or genetic testing), the subtype of the disease, date of visit, current age, physical data, liver span and biochemical findings, including blood glucose levels, triglyceride, cholesterol, uric acid, lactate, vitamin D level, neutrophil count and haematocrit (HCT).

### 2.8. Blood Samples Collection

By the end of the check-up visit, physicians at QRAH ordered the withdrawal of some blood samples to analyse glucose, cholesterol, triglyceride, uric acid, lactic acid, vitamin D level and complete blood count (CBC) (to check neutrophils count and HCT) with a maximum of three hours fasting. Biochemical variables were assessed under the same conditions in all patients. It should be noted that the physicians’ order to check neutrophils count, which is used to distinguish between type 1a and 1b, was not applied by any researchers cited in this manuscript except in one paper by Santos et al. [36], who said that low levels of neutrophils are specifically related to type 1b. These data were gathered at baseline (before MCT oil intervention) and three months later during follow-up visits.

### 2.9. Statistical Analyses

The Statistical Package for Social Sciences software (SPSS, version 20, Chicago. Inc., Chicago, Illinois, USA) was used for data processing and data analysis. Characteristics of subjects’ variables were described using frequency distribution for categorical variables and the mean and standard deviation for continuous variables. The normality assumption was checked, but all of the variables were not normally distributed, nor did they attain normal distribution after logarithmic transformation (except the glucose variable). Comparisons were made between paired groups with paired samples, including a *t*-test (for parametric variables) and a Wilcoxon-signed ranks test (for non-parametric variables). The McNemar test (for dichotomous dependent variable) and the marginal homogeneity test (for more than two categories) were used to evaluate the effectiveness of dietary recommendation and MCT oil intake in the same intervention group in addition to an independent *t*-test to determine whether there is a statistically significant difference between the means in two unrelated groups. A *p*-value of <0.05 was considered the cut-off level for statistical significance.

## 3. Results

### 3.1. Socio-Demographic Characteristics of the Study Population

Thirty-eight patients were included in this study. The median age was 6.4 years, ranging from 1.5 to 15.5 years old. According to the National Organization for Rare Disorders (NORD), the critical initial presentation of hypoglycaemia usually appears between three and four months or immediately after birth. In this study, the age at diagnosis was from birth up to 48 months (median = 5 months). The patient who was diagnosed the latest had an abdominal distention and short stature at four years old. This sheds light on a serious point: physicians’ poor knowledge of rare inherited metabolic disorders, including GSD-1, may lead to delayed diagnosis and, subsequently, the onset of irreversible symptoms, such as short stature. Results showed that 20 patients (52.6%) were male, and 18 patients (47.4%) were female. Regarding the consanguinity degree, 26 patients (68.4%) parents were first-degree relatives, nine (23.7%) were second-degree relatives, and three (7.9%) were not relatives. In this study, the degree of consanguinity was not stated as a risk factor, but our results revealed that parents with first-degree consanguinity had a higher chance of having a child with GSD, particularly since the inheritance of this condition is autosomal recessive. 

### 3.2. Laboratory Results Analysis and Abdominal Ultrasound

As shown in Table 2 and Table 3, the biochemical tests presented show that all results were significantly improved after dietary recommendations and the use of MCT oil, including serum glucose, triglycerides, cholesterol, lactic acid, uric acid, HTC and neutrophils.

All patients underwent abdominal ultrasound for the assessment of liver span (except for two patients). The liver span was improved moderately during the study. There was a statistically significant reduction in the liver span from (16.01 ± 2.652 cm) to (14.85 ± 2.256 cm) (*p* < 0.0001). (Note: a normal or enlarged liver span was classified based on the criteria set by the health facility where the study had been conducted.)

### 3.3. Nutritional Behaviours of the Study Population

The whole study population (100%) visited the nutritional clinic at QRAH and followed their classic GSD diet. Moreover, all the children cared about food quality and had good nutritional knowledge. The nutritional guidelines were explained in detail during every visit to the clinic, and parents were encouraged to voice any queries or concerns they had to ensure their thorough understanding of and their children’s firm adherence to the recommended diet to help them avoid any complications of the disease, thus guaranteeing a good quality of life. Parents were asked to record what their children consumed between visits, and the dietitian discussed the diet components with them, checking if recommendations had been followed.

### 3.4. Medical Information and Other GSD-Related Issues

Regarding the medical information and other GSD-related issues, Table 1 illustrates that 89.5% of the patients were taking multivitamins. It was mentioned previously that the diet schedule for GSD-1 patients is not nutritionally well-balanced regarding the amounts of certain vitamins and minerals. Therefore, it was recommended that they consume multivitamins and minerals, especially calcium and vitamin D [2]. Accordingly, 60.5% were vitamin D deficient, particularly before starting the clinical trial. Therefore, they were advised to obtain vitamin D supplements as prescribed by the metabolic physician based on their vitamin D levels. Worldwide, 80% of patients with GSD are classified as type 1a, and 20% are classified as type 1b. Surprisingly, most of our patients (27 out of 38; 71.1%) in Jordan were classified as type 1b and the rest as type 1a (11 out of 38; 28.9%). Of the study population, 68.4% were hypoglycaemic when initial clinical presentation was observed.

Regarding the method of diagnosis, it was stated earlier that the method of choice for differential diagnosis of GSD is genetic testing. Yet, due to the high cost of the test and the financial status of parents, access to genetic testing is very limited, which is why only 18.4% (7 out of 38) of the study population had undergone genetic testing (those with a positive family history and/or clinical presentation). 

### 3.5. Anthropometric Measurements and Body Composition Analysis

#### 3.5.1. Anthropometric Measurements

Growth parameters, including weight-for-age, height-for-age and BMI-for-age, were described in this section. The percentiles’ classifications were provided by the 2006 WHO growth charts for children aged < 2 years and the CDC growth charts for children aged ≥ 2 years. According to BMI Calculator Child and Teen, 2019, all children’s percentiles were obtained and analysed before and after the MCT oil intervention. Significant improvement in height-for-age for patients aged ≥ 2 years (*p* = 0.034) was observed (Table 4).

#### 3.5.2. Body Composition Analysis

**Figure 1:**
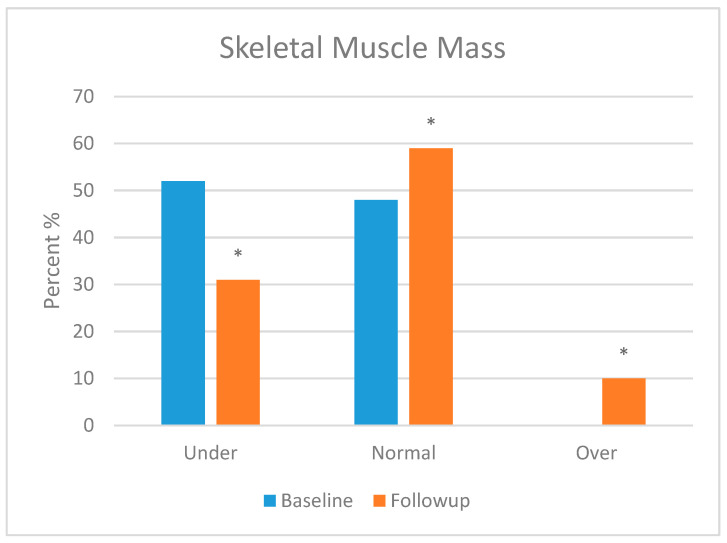
Skeletal muscle mass before and after medium-chain triglyceride (MCT) oil intervention (*p* = 0.002). * indicates significant differences between baseline and follow up.

**Figure 2:**
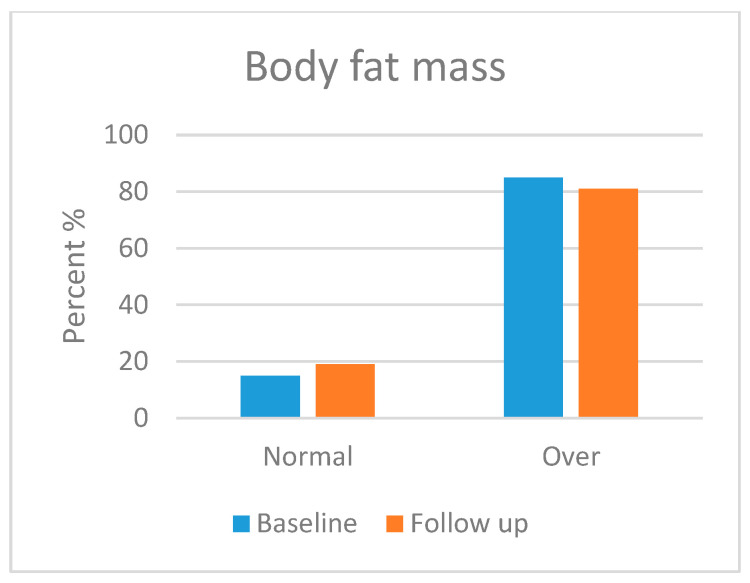
Body fat mass before and after medium-chain triglyceride (MCT) oil intervention (*p* = 0.989).

**Figure 3:**
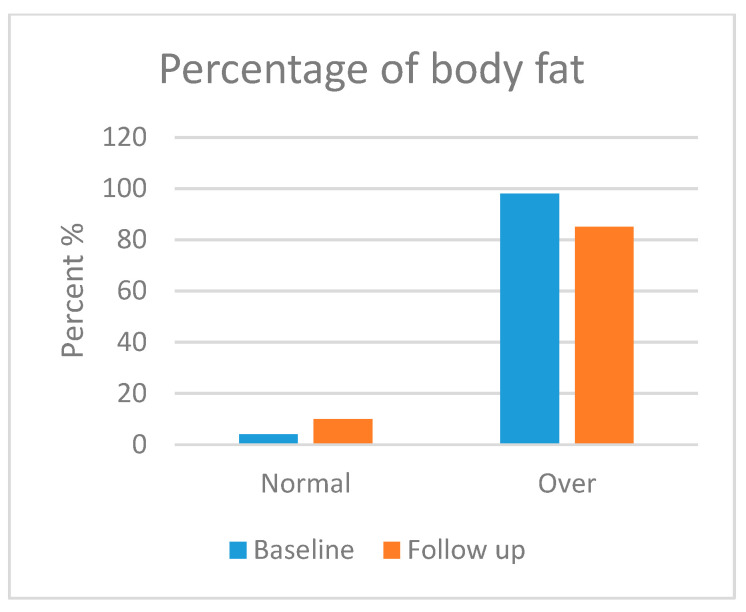
Percentage of body fat before and after medium-chain triglyceride (MCT) oil intervention (*p* = 0.250).

**Figure 4:**
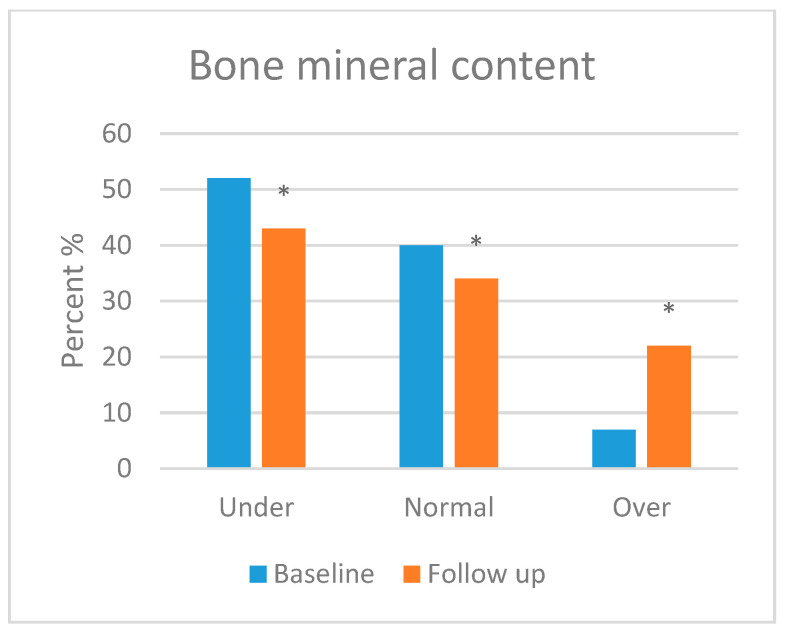
Bone mineral content before and after medium-chain triglyceride (MCT) oil intervention (*p* = 0.011). * indicates significant differences between baseline and follow up.

Skeletal muscle mass (SMM), body fat mass (BFM), percentage body fat (PBF) and bone mineral content (BMC) were assessed in this section. Body composition was analysed in most of the participants, but three patients refused to take the follow-up test. Moreover, three patients were excluded from the test due to their age (<3 years). Figure 1, Figure 2, Figure 3 and Figure 4 show the body composition analysis before and after MCT oil intervention. As seen in Figure 1 and Figure 4, SMM and BMC had improved significantly after MCT oil intervention (*p* = 0.002 and *p* = 0.011, respectively), whereas, as seen in Figure 2 and Figure 3, there were no significant differences between BFM and PBF after MCT oil intervention (*p* = 0.989 and *p* = 0.250, respectively). This was the first study that evaluated the BMC using the InBody^®^ test for patients with GSD-1 who were on the classic diet along with MCT oil intervention. 

## 4. Discussion

GSD type 1 is caused by a deficiency of either glucose-6-phosphatase (GSD type 1a) or glucose-6-phosphate translocase (G6PT) (GSD type 1b), which disrupts the process of glycogenolysis and gluconeogenesis. Eventually, this leads to fasting hypoglycemia, the hallmark of the disease [8,13,26,36].

In response to hypoglycaemia, secondary biochemical abnormalities arose. Herein, glycogenolysis provides high amounts of glucose 6 phosphatase (G6P) that act in the following way.

- First, as a substrate for the pentose phosphate pathway (PPP), which, in turn, increases the catabolic process of ribose-5-phosphate to produce uric acid, causing hyperuricemia, which may predispose, if left untreated, to gout.
- Second, this greater flux of G6P enters the glycolytic pathway to produce huge amounts of pyruvate, leading to an alternative route that produces lactate under the action of lactate dehydrogenase (LDH), which results in lactic acidosis (hyperlactatemia).
- Third, pyruvate enters the Krebs cycle and produces acetyl CoA. Then, the latter starts lipogenesis, i.e., forming cholesterol and fatty acids. These fatty acids bind to glycerol (from glycolysis) to synthesise triglycerides, thus leading to hyperlipidaemia [42], as well as the production of malonyl CoA, which inhibits the key enzyme in β oxidation, carnitine palmitoyl transferase I (CPT I) [14,43].

For GSD-1b, recent demonstrations have concerned the mechanism of neutropenia. Burda and Hochuli [13], Chou et al. [44] and Jun et al. [45] referred to dysfunctional G6PT, which decreases the amount of glucose produced and triggers the stress process leading to apoptosis and neutrophil death within the ER. MCT is directly absorbed into the portal vein with the least machinery, with no need for bile salts or pancreatic enzymes and without being included in chylomicrons [46,47]. In a straight line, MCT enters the mitochondrial matrix without depending on the carnitine shuttle system that is required to transfer the long-chain fatty acids (LCFA) across the mitochondrial membrane for the event of β-oxidation so that it can bypass the metabolic block of LCFA [46,47,48]; therefore, suppressing de novo lipogenesis leads to decreased levels of TG and cholesterol [16,46]. Concentrations of TG decreased, but most of them stayed at higher levels. This may be due to the limitation of our study, specifically a limited follow-up duration. Those patients need more time on the diet schedule along with the MCT intake to obtain the best results.

Experiments on MCT oil were performed in 2007 by a group of researchers from Japan [48], who concluded that MCT slows glucose uptake and maintains euglycemia. This was also declared by Derks and van Rijn [14]. In other words, MCT decelerates the uptake of glucose, so it is resistant to the hypoglycaemic episodes caused by compromised glycogenolysis and gluconeogenesis. Our results are in complete agreement with studies published by Das et al. [18] and Nagasaka et al. [48] and find that if glucose levels become normalised, then the rest of the biochemical derangements underlying GSD-1 will be controlled. Herein, glycolysis is inhibited, and reasonable lactate levels are reached. It is worth recalling that hyperuricemia is not merely due to the high production of uric acid but also to the decreased renal clearance as a result of competitive inhibition caused by hyperlactatemia [16].

Concerning HCT, our results are quite similar to those of Kishnani et al. [8], who state that Rake et al. [29] performed a study on 32 paediatric patients with GSD-1a, where 17–60% were anaemic. Another study in the same article mentions that 72% with GSD-1b were also anaemic. It should be pointed out that anaemia is caused by several factors, including persistent hyperlactatemia, dietary protocol, bleeding diathesis, presence of liver adenoma, IBD, renal insufficiency and poor metabolic control.

Our findings are in contrast to those mentioned by Melis et al. [49], who concluded that good metabolic control does not affect neutrophil count. It is worth noting that the majority of our patients were classified as type 1b (27 out of 38), and neutropenia is specifically associated with them. Accordingly, it is expected to find that 60.5% of the patients are neutropenic. Moreover, the results ensured that preventing hypoglycaemia that initiates a long cascade of metabolic derangements, including increased liver span, can lead to outstanding reduction [7,13,31,36].

There were no significant differences in the mean percentile value of weight-for-age for all children, height-for-age for patients aged <2 years or BMI-for-age for patients aged <2 years. This lack of significance may be due to the short duration of the study. Therefore, a longer period of study is required to obtain significant improvements. All patients had a slight improvement in height, with the rate of increase equal to 1.87 cm. Although patients’ height had increased, it was still within the lower limits. These findings are in agreement with Das et al. [18] and further support the role of MCT in growth and development. Moreover, it was established by Santos et al. [36] that keeping controlled metabolic profiles early in life, especially with a view to preventing hypoglycaemia, will improve the growth process, and good height for age can be obtained.

In terms of body composition, first, SMM was statistically improved, which is consistent with a previous report [18] that stated that good metabolic control can maintain good growth and development. Second, BMC was also statistically improved; several factors attributed to low bone mineral content had been stated by Dambska et al. [26], including low intake of vitamin D and foods containing calcium (due to the restricted nature of the GSD diet) and chronic hypoglycaemia, which leads to both hyperlactatemia and hypercortisolemia. A mechanism described by Minarich et al. [24] demonstrated that persistent hypoglycaemia enhances the production of glucagon, which activates the production of cyclic adenosine monophosphate (cAMP) via G-protein-coupled receptors, resulting in elevated levels of cellular G6P. As mentioned previously, this G6P shunts its direction to an alternative pathway, resulting in de novo lipogenesis and hyperlactatemia. The latter, in turn, increases bone resorption and decreases bone formation (enhancing the production of osteoclast and inhibiting the production of osteoblast). Eventually, this leads to decreased bone mineralisation. Many studies, such as Das et al. [18] and Minarich et al. [24], agreed that good metabolic control, along with supplementation of calcium and vitamin D, can sustain good bone health. In contrast, BFM and PBF had no significant improvement during the time of the study, although Li et al. [46], Shah and Limketkai [47] and Tanchoco et al. [50] mention that MCT enhances the process of fat breakdown in the adipose tissue. This insignificance may be due to the limited time frame of the study. Both parameters may need more time to be improved. It is noted that our results support the hypothesis that dietary and MCT oil intervention improves the metabolic profile and growth parameters of children patients with GSD type 1.

## 5. Limitations

A few limitations of this study are the small sample size due to the rarity of the disease, the lack of a control group, the limited duration of the follow-up and the limited access to and high cost of genetic testing due to financial issues of the guardians in the study population.

## 6. Conclusions

In this study, which is the first such study in Jordan, unlike the worldwide trend, type 1b was the most common type of GSD-1. The life-long dietary management of GSD type 1 is the cornerstone in controlling signs and symptoms and decreasing the onset rate of complications of the disease. One of the most critical symptoms is fasting hypoglycaemia, which initiates a cascade of biochemical and clinical abnormalities leading to long-term complications. Many of these abnormalities, such as chronic hypoglycaemia, persistent hypertriglyceridemia, hypercholesterolemia, lactic acidosis and hyperuricemia, partially respond to the traditional GSD diet. Therefore, we studied the effect of MCT oil along with the prescribed diet, which resulted in outstanding outcomes in ameliorating biochemical results, such as normalising serum glucose, lactate and uric acid and a significant reduction in concentrations of TG and cholesterol, as well as a slight drop in liver span. In addition to a remarkable improvement in biochemical tests, body composition, including skeletal muscle mass and bone mineral content, were also improved after the intervention. Early diagnosis and routine follow-up visits are essential to keep metabolic control as optimal as possible.

## Figures and Tables

**Table 1:** Medical information and glycogen storage disease (GSD)-related characteristics of the patients.

Variable	N	%
Multivitamins intake		
Yes	34	89.5
No	4	10.5
Vitamin D deficiency		
Yes	23	60.5
No	15	39.5
Frequency and dose of vitamin D intake		
None	2	5.3
Daily (2000 IU *)	10	26.3
Every other day (5000 IU)	6	15.8
Weekly (50,000 IU)	20	52.6
Calcium supplement intake		
Yes	22	57.9
No	16	42.1
GSD subtype		
Type 1a	11	28.9
Type 1b	27	71.1
Initial clinical symptoms		
Hypoglycaemia	26	68.4
Hepatomegaly	9	23.7
Both	3	7.9
Method of diagnosis		
Clinical presentation	15	39.5
Liver biopsy and clinical presentation	9	23.7
Clinical presentation and positive family history	7	18.4
Genetic testing and positive family history	4	10.5
Genetic testing and clinical presentation	3	7.9

* IU: international unit, N: frequency.

**Table 2:** Laboratory results before and after medium-chain triglyceride (MCT) oil intervention in patients.

	MCT Intervention	Mean	Normal Range	SD	Minimum	Maximum	Median	*p*-Value
Glucose (mg/dL)	Before After	53.03 90.47	70–110	16.619 18.569	13 58	93 142	55.50 88.00	≤0.001 b
TG (mg/dL)	Before After	657.16 368.35	50–200	660.812 255.958	202.6 126.9	2895.0 1136.8	351.85 284.60	≤0.001 a
Cholesterol (mg/dL)	Before After	251.16 170.21	70–200	73.486 65.876	122 103	405 383	249.50 143	≤0.001 a
Uric Acid (mg/dL)	Before After	7.70 5.94	2.4–7.0	1.917 1.660	5.56 2.7	13.3 12.70	7.10 5.85	≤0.001 a
Lactate (mg/dL)	Before After	53.17 19.51	0–25.2	27.030 6.533	13.9 10.11	129.00 40.40	41.00 18.05	≤0.001 a
Neutrophils Count (103/µL)	Before After	1.93 1.82	1.5–7.0	1.802 1.145	0.10 0.22	6.19 4.42	1.06 1.5	0.331 a
HCT (%)	Before After	31.73 32.62	31–54	4.580 4.748	22.1 17.2	42.1 47.6	31.25 32.15	0.020 a

a. Wilcoxon signed ranked test; b. Student’s *t*-test; SD: standard deviation; TG: triglyceride; HCT: haematocrit; Reference ranges: glucose, 70–110 mg/dL at age > 3 days; TG, 50–200 mg/dL; cholesterol, 70–190 mg/dL at age 1–2 years, 135–200 mg/dL at age 2–15 years, 120–200 mg/dL at age > 15 years; uric acid, 2.4–5.4 mg/dL at age < 10 years, 2.7–5.9 mg/dL at age 10–11 years, 3.1–6.4 mg/dL at age 11–12 years, 3.4–6.9 mg/dL at age 12–13 years, 3.7–7.4 mg/dL at age 13–14 years, 3.4–7.0 mg/dL at age > 14 years; lactate, 0–25.2 mg/dL; neutrophil count, 1.5–8.5 103/µL at age 6 months–4 years, 1.8–8.0 103/µL at age 4–13 years, 2–7 103/µL at age > 13 years; HCT, 31–37% at age 6 months–4 years, 37–43% at age 4–13 years, 40–54% at age > 13 years.

**Table 3:** Enhancement of abnormal biochemical tests after dietary recommendations and medium-chain triglyceride (MCT) oil in patients.

Laboratories Tests	Abnormal Levels before MCT Oil (%)	Abnormal Levels after MCT Oil (%)	*p*-Value
Glucose (mg/dL)	94	7.9	0.001
Lactic Acid (mg/dL)	94.7	18.4	0.001
Triglycerides (mg/dL)	100	71.1	0.001
Cholesterol (mg/dL)	73.7	21.1	0.001
Uric Acid (mg/dL)	97.4	52.6	0.001
Neutrophils (103/µL)	60.5	55.3	0.001

**Table 4:** Anthropometric measurements of patients before and after receiving medium-chain triglyceride (MCT) oil therapy.

Variable		Baseline		Follow-Up		
	Age Group	Mean	SD	Mean	SD	*p*-Value
Weight-for-age	<2 years ≥2 years	36.67 25.68	41.826 29.014	39.17 26.14	48.008 30.101	0.593 a 0.940 a
Height-for-age	<2 years ≥2 years	33.27 4.13	46.708 8.351	33.13 5.25	36.258 10.328	1.00 a 0.034 a
BMI-for-age	<2 years ≥2 years	45.53 75.34	27.632 20.487	62.90 71.63	42.062 23.503	0.593 a 0.074 a

a. Independent *t*-test; BMI: body mass index; SD: standard deviation.

## Data Availability

The original contributions presented in the study are included in the article, further inquiries can be directed to the corresponding author.
